# Identification of *dfrA14* in two distinct plasmids conferring trimethoprim resistance in *Actinobacillus pleuropneumoniae*

**DOI:** 10.1093/jac/dkv121

**Published:** 2015-05-08

**Authors:** Janine T. Bossé, Yanwen Li, Stephanie Walker, Tom Atherton, Roberto Fernandez Crespo, Susanna M. Williamson, Jon Rogers, Roy R. Chaudhuri, Lucy A. Weinert, Olusegun Oshota, Matt T. G. Holden, Duncan J. Maskell, Alexander W. Tucker, Brendan W. Wren, Andrew N. Rycroft, Paul R. Langford

**Affiliations:** 1 Section of Paediatrics, Department of Medicine, Imperial College London, St Mary's Campus, London W2 1PG, UK; 2 Animal and Plant Health Agency (APHA) Bury St Edmunds, Rougham Hill, Bury St Edmunds, Suffolk IP33 2RX, UK; 3 Department of Veterinary Medicine, University of Cambridge, Madingley Road, Cambridge CB3 0ES, UK; 4 The Wellcome Trust Sanger Institute, Wellcome Trust Genome Campus, Hinxton, Cambridge CB10 1SA, UK; 5 Faculty of Infectious and Tropical Diseases, London School of Hygiene & Tropical Medicine, Keppel Street, London WC1E 7HT, UK; 6 Department of Pathology and Pathogen Biology, The Royal Veterinary College, Hawkshead Campus, Hatfield, Hertfordshire AL9 7TA, UK

**Keywords:** animal infections, antibiotic resistance, respiratory tract

## Abstract

**Objectives:**

The objective of this study was to determine the distribution and genetic basis of trimethoprim resistance in *Actinobacillus pleuropneumoniae* isolates from pigs in England.

**Methods:**

Clinical isolates collected between 1998 and 2011 were tested for resistance to trimethoprim and sulphonamide. The genetic basis of trimethoprim resistance was determined by shotgun WGS analysis and the subsequent isolation and sequencing of plasmids.

**Results:**

A total of 16 (out of 106) *A. pleuropneumoniae* isolates were resistant to both trimethoprim (MIC >32 mg/L) and sulfisoxazole (MIC ≥256 mg/L), and a further 32 were resistant only to sulfisoxazole (MIC ≥256 mg/L). Genome sequence data for the trimethoprim-resistant isolates revealed the presence of the *dfrA14* dihydrofolate reductase gene. The distribution of plasmid sequences in multiple contigs suggested the presence of two distinct *dfrA14*-containing plasmids in different isolates, which was confirmed by plasmid isolation and sequencing. Both plasmids encoded mobilization genes, the sulphonamide resistance gene *sul2*, as well as *dfrA14* inserted into *strA*, a streptomycin-resistance-associated gene, although the gene order differed between the two plasmids. One of the plasmids further encoded the *strB* streptomycin-resistance-associated gene.

**Conclusions:**

This is the first description of mobilizable plasmids conferring trimethoprim resistance in *A. pleuropneumoniae* and, to our knowledge, the first report of *dfrA14* in any member of the Pasteurellaceae. The identification of *dfrA14* conferring trimethoprim resistance in *A. pleuropneumoniae* isolates will facilitate PCR screens for resistance to this important antimicrobial.

## Introduction

*Actinobacillus pleuropneumoniae* causes porcine pleuropneumonia, an economically important endemic disease that can be difficult to control.^1^ Good husbandry practices and vaccination can help to reduce the incidence of acute disease, and the early use of effective antimicrobials is essential to limit its spread and severity. A knowledge of the antimicrobial susceptibility patterns of *A. pleuropneumoniae* is important so that informed treatment decisions can be made.

In the UK, the most commonly used antimicrobials for the treatment of food animals (86% of which are used for pigs and poultry) are tetracyclines, β-lactams and trimethoprim/sulphonamides.^2^ Sulphonamides have been widely used since the 1930s for the treatment of both human and veterinary diseases.^3,4^ Trimethoprim, introduced in the 1960s, is often coadministered with sulphonamides.^3^

Resistance to both trimethoprim and sulphonamides can be mediated either by mutations in the chromosomally encoded target enzymes (dihydropteroate synthase and dihydrofolate reductase, respectively) or by the acquisition of transferable genes encoding alternative drug-insensitive enzymes.^3,4^ There are three known genes encoding alternative dihydropteroate synthases (*sul1*, *sul2* and *sul3*)^5^ and >30 *dfr* genes encoding trimethoprim-insensitive dihydrofolate reductases.^6^

Sulphonamide resistance conferred by *sul2*, carried on small plasmids, has been reported for *A. pleuropneumoniae*^7–9^ and other Pasteurellaceae.^10^ However, little is known regarding the genetic basis of trimethoprim resistance in the Pasteurellaceae. Single bovine and porcine isolates of *Pasteurella multocida*^11^ and *Pasteurella aerogenes*^12^ have harboured plasmids carrying *dfrA20* and *dfrA1*, respectively, whereas trimethoprim-resistant *Haemophilus influenzae* has been shown to have mutations in the chromosomally encoded dihydrofolate reductase.^13^

In this study, we have identified the genetic basis of trimethoprim resistance in *A. pleuropneumoniae* using WGS followed by plasmid isolation and confirmatory sequencing. Two distinct plasmids carrying *dfrA14* were found, the first known description of this gene in the Pasteurellaceae.

## Materials and methods

### Bacterial strains and antimicrobial resistance testing

A total of 106 clinical isolates of *A. pleuropneumoniae*, cultured from the pneumonic lungs of pigs submitted for diagnostic investigation to the then Animal Health and Veterinary Laboratory Agency (now Animal and Plant Health Agency) diagnostic laboratories in England between 1998 and 2011, were selected for study. The majority of isolates were from 2005–10 (20, 26, 11, 12, 14 and 8 isolates, respectively), with none from 2000–01 and only 1–4 from each of the other years. Serovars 2 (11%), 6 (6.5%), 7 (8.5%), 8 (72.0%) and 12 (2.0%) were represented, reflecting the serovar distribution in the UK.^14^ *A. pleuropneumoniae* MIDG2331 is a plasmid-free serovar 8 clinical isolate that was cultured from pneumonic pig lungs in 1995. MIDG2331 was made NAD-independent by the chromosomal insertion (replacing part of *ureC*) of the *Haemophilus ducreyi nadV* gene, yielding MIDG2331Δ*ureC*::*nadV*.^15^ All the strains were grown at 37°C with 5% CO_2_ on brain heart infusion (BHI; Difco) agar supplemented with 0.01% NAD and, when required, with trimethoprim (10 mg/L).

For all isolates, MICs were determined for trimethoprim and sulfisoxazole by agar dilution susceptibility testing, according to the CLSI M31-A3 guidance.^16^

### Genome sequencing and analysis

Genomic DNA was extracted from the 16 trimethoprim-resistant (MIC >32 mg/L) *A. pleuropneumoniae* isolates (Table 1) using the FastDNA Spin Kit (MP Biomedicals), according to the manufacturer's protocol for bacterial cells, and 0.5 μg was used for library preparation and sequencing as previously described.^17^

**Table 1.** Genes identified by ResFinder in *A. pleuropneumoniae* isolates from the UK with resistance to trimethoprim and sulfisoxazole

MIDG number	Year	Location	Serovar	Trimethoprim (mg/L)	Sulfisoxazole (mg/L)	*dfrA14*^a^		*sul2*^b^		*strA*^c^		*strB*^d^	
						contig	length	contig	length	contig	length	contig	length
2356	1998	Bury St Edmunds	7	>32	>512	6	3451	6	3451	6	3451	6	3451
2657	2005	Winchester	8	>32	>512	31	1757	26	943	31	1757		
2664	2005	Bury St Edmunds	8	>32	>512	65	1421	55	943				
3346	2005	Thirsk	8	>32	>512	48	1757	57	943	48	1757		
3201	2006	Bury St Edmunds	8	>32	>512	10	1765	28	951	10	1765		
3221	2006	Bristol	8	>32	256	20	1761	54	947	20	1761		
3349	2006	Thirsk	8	>32	>512	47	1421	60	943				
3224	2007	Bury St Edmunds	8	>32	>512	49	3429	49	3429	49	3429	49	3429
3232	2007	Thirsk	8	>32	>512	12	1761	20	947	12	1761		
3370	2009	Thirsk	8	>32	>512	57	1759	106	945	57	1759		
3371	2009	Thirsk	8	>32	>512	50	1759	95	945	50	1759		
3372	2009	Thirsk	8	>32	>512	45	1759	102	945	45	1759		
3378	2009	Bury St Edmunds	8	>32	>512	56	1753	15	4128	56	1753		
3388	2009	Thirsk	8	>32	>512	6	1777	22	963	6	1777		
3389	2009	Thirsk	8	>32	>512	30	1610	16	961	30	1610		
3395	2010	Thirsk	8	>32	>512	5	636	26	963	74	442		

^a^99.8% identity (483/483 bp) with *dfrA14* from *Salmonella enterica* subsp. *enterica* serovar Typhimurium (DQ388123).

^b^100% identity (816/816 bp) with *sul2* from *Acinetobacter bereziniae* (GQ421466).

^c^100% identity (529/804 bp) with *strA* from a *Shigella flexneri* plasmid (AF321551) for MIDG2356 and MIDG3224, and 99.8% identity (529/804 bp) with *strA* from an *Erwinia amylovora* plasmid (M96392) for all others with *strA* (NB: in MIDG3395 only 512/804 bp of the gene were detected).

^d^99.9% identity (705/837 bp) with *strB* from an *Erwinia amylovora* plasmid (M96392).

ResFinder (www.genomicepidemiology.org) was used to identify acquired antimicrobial resistance genes (using a threshold of 98% identity) in the draft genomes. Contigs identified by ResFinder (Table 1) have been submitted to GenBank (accession numbers: contig006_MIDG2356 =KP196974; contig026_MIDG2657 = KP196975; contig031_MIDG2657 =KP196976; contig055_MIDG2664 = KP196977; contig065_MIDG2664 =KP196978; contig010_MIDG3201 = KP196979; contig028_MIDG3201 =KP196980; contig020_MIDG3221 = KP196981; contig054_MIDG3221 =KP196982; contig049_MIDG3224 = KP196983; contig012_MIDG3232 =KP196984; contig020_MIDG3232 = KP196985; contig048_MIDG3346 =KP196986; contig057_MIDG3346 = KP196987; contig047_MIDG3349 =KP196988; contig060_MIDG3349 = KP196989; contig106_MIDG3370 =KP196990; contig050_MIDG3371 = KP196991; contig095_MIDG3371 =KP196992; contig045_MIDG3372 = KP196993; contig102_MIDG3372 =KP196994; contig015_MIDG3378 = KP196995; contig056_MIDG3378 =KP196996; contig006_MIDG3388 = KP196997; contig022_MIDG3388 =KP196998; contig016_MIDG3389 = KP196999; contig030_MIDG3389 =KP197000; contig005_MIDG3395 = KP197001; contig026_MIDG3395 =KP197002; and contig074_MIDG3395 = KP197003).

### Isolation and characterization of plasmids

Plasmids were extracted from *A. pleuropneumoniae* isolates MIDG3224 and MIDG3389, selected as representing two different patterns of resistance genes identified by ResFinder (Table 1), using the QIAprep Spin Miniprep kit (Qiagen). Attempts were made to transform plasmids into *Escherichia coli* Stellar cells (Clontech) by heat shock, with selection on LB agar containing trimethoprim (10 or 20 mg/L). The conjugal transfer of plasmids from MIDG3224 and MIDG3389 into MIDG2331Δ*ureC*::*nadV* was carried out as previously described,^18^ with transconjugants selected on BHI agar (without NAD) supplemented with 10 mg/L trimethoprim.

The MICs of trimethoprim and sulfisoxazole were determined for selected trimethoprim-resistant transconjugants, as described above, and the presence of *dfrA14*, *sul2* and *nadV* was determined by QiagenFast PCR (Qiagen) using primer pairs dfrA14_for (CATTGATAGCTGCGAAAGCGAAAAACGGC)/dfrA14_rev (ATCGTCGATAAGTGGAGCGTAGAGGC), sul2_for (TCAACATAACCTCGGACAGTTTCTC)/sul2_rev (GGGAATGCCATCTGCCTTGAGC) and nadV_for (CTAGTAACCGAGCCCGCCTAATGAG)/nadV_rev (GGCGGCCGCACTAGTGATTACAAG).

The complete sequences of plasmids pM3389T and pM3224T, isolated from transconjugants, were determined using a primer walking strategy (GenBank accession numbers pM3224T = KP197004 and pM3389T =KP197005). These sequences were subsequently used to search the draft genomes of the remaining trimethoprim-resistant isolates using BLASTn.

## Results and discussion

Trimethoprim resistance (MIC >32 mg/L) was detected in 16 out of 106 *A. pleuropneumoniae* isolates, and all 16 were resistant to sulfisoxazole (MIC ≥256 mg/L) (Table 1). A further 32 isolates were resistant to sulfisoxazole only (data not shown), which is not surprising given that trimethoprim, often coadministered with sulphonamides, was introduced for use 30 years after sulphonamides. Co-resistance to trimethoprim and sulfisoxazole was found in serovar 7 and 8 isolates obtained from four different geographical locations in England as early as 1998 (1 out of 3 isolates), with the largest proportion identified in 2009 (6 out of 14 isolates).

ResFinder analysis (Table 1) of the draft genomes identified the trimethoprim resistance gene *dfrA14* on contigs ranging from 636 to 3451 bp in all trimethoprim-resistant isolates. In all but two isolates (MIDG2664 and MIDG3349) a partial *strA* gene was identified on the same contig as *dfrA14*, and in two isolates (MIDG2356 and MIDG3224) *sul2* and *strB* were also found on the same contig as *dfrA14*. The *sul2* gene was identified on separate small contigs (768–963 bp) in all other isolates. BLASTx analysis of the *dfrA14*-containing contigs of MIDG2664 and MIDG3349 revealed partial *strA* sequences flanking the *dfrA14* gene in both cases. Furthermore, alignments of the *dfrA14*-containing contigs showed that the *strA*5′-*dfrA14*-*strA*3′ sequences were identical in all 16 isolates, although the shorter contigs in MIDG2664 and MIDG3349 were missing the first 205/529 bp of the *strA*5′ sequence, which was not detected by ResFinder. Alignments of the *dfrA14*-containing contigs also suggested two different trimethoprim resistance plasmids: contigs from MIDG2356 and MIDG3224 were identical over the 3429 bp common to both, and contigs from the remaining isolates showed 100% identity where alignment was possible, given the different lengths of the contigs.

**Figure 1.**
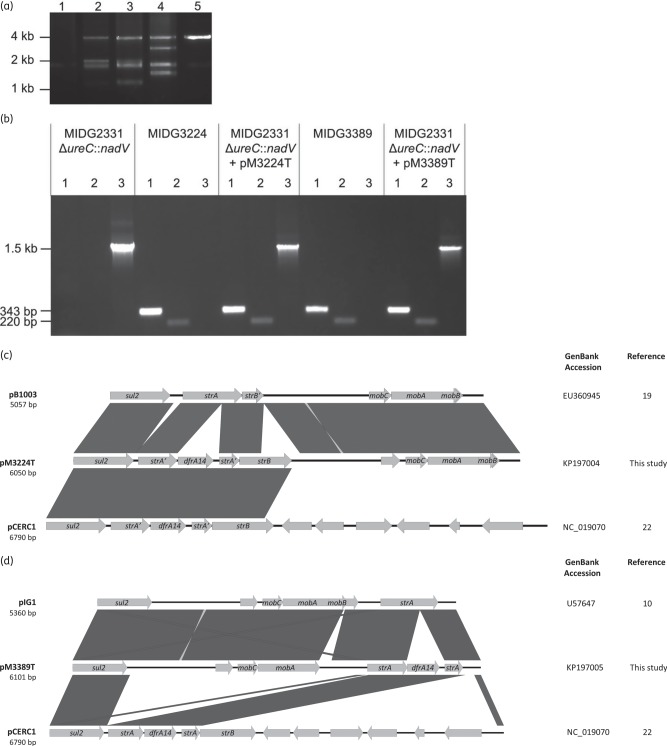
Isolation and characterization of newly identified *dfrA14*-containing *A. pleuropneumoniae* plasmids. (a) Comparison of plasmid extracts from MIDG2331Δ*ureC*::*nadV* (Lane 1), conjugal donor strains (Lane 2 = MIDG3224 and Lane 4 = MIDG3389) and respective trimethoprim-resistant transconjugants, showing the transfer of plasmids (Lane 3 = pM3224T and Lane 5 = pM3389T) into MIDG2331Δ*ureC*::*nadV*. (b) PCR amplification of *dfrA14* (343 bp amplicon; Lane 1 in each section), *sul2* (220 bp amplicon; Lane 2 in each section) and *nadV* (1.5 kb amplicon; Lane 3 in each section) from MIDG2331Δ*ureC*::*nadV*, MIDG3224, MIDG2331Δ*ureC*::*nadV*+pM3224T, MIDG3389 and MIDG2331Δ*ureC*::*nadV*+pM3389T, as indicated for each section of the gel. (c) Schematic comparison of pM3224T with the most closely related Pasteurellaceae plasmid, pB1003, and pCERC1, a *dfrA14*-containing plasmid found in Enterobacteriaceae. (d) Schematic comparison of pM3389T with the most closely related Pasteurellaceae plasmid, pIG1, and pCERC1. Reading frames are indicated by arrows, with arrowheads showing the direction of transcription; only relevant genes have been annotated (*sul2*: sulphonamide resistance; *strA*, *strB*: streptomycin resistance; *dfrA14*: trimethoprim resistance; *mobA*, *mobB*, *mobC*: plasmid mobilization; *strB′*: partial *strB*; *strA′*: partial *strA*). Dark grey blocks between sequences indicate ≥99% nucleotide sequence identity.

The distribution of sequences among the small contigs suggested the possibility of multiple plasmids sharing common sequences. No known plasmids were detected in the draft genomes using PlasmidFinder (www.genomicepidemiology.org), but an analysis of the endogenous plasmid profiles for MIDG3224 and MIDG3389 suggested multiple plasmids, at least in the latter (Figure 1a). Conjugal transfer from MIDG3224 and MIDG3389 into a plasmid-free recipient strain (MIDG2331Δ*ureC*::*nadV*) was used to isolate trimethoprim resistance plasmids pM3224T and pM3389T prior to complete nucleotide sequencing (Figure 1a). Trimethoprim-resistant transconjugants were positive for *dfrA14* and *nadV* by PCR, indicating the successful mobilization of plasmids from MIDG3224 and MIDG3389 into MIDG2331Δ*ureC*::*nadV* (Figure 1b). Furthermore, the amplification of *sul2* sequences from transconjugants suggested that both pM3224T and pM3389T also encode sulphonamide resistance (Figure 1b), and the MICs of trimethoprim and sulfisoxazole were the same for the transconjugants as for the donor strains. A primer walking strategy was used to determine the complete nucleotide sequences of pM3224T and pM3389T as representatives of the two different trimethoprim resistance plasmids indicated above.

Plasmid pM3224T (6050 bp) was found to share the greatest similarity (99% identity with 81% coverage) with pB1003 (accession no. EU360945) isolated from *P. multocida* from pigs in Spain^19^ (Figure 1c). These two plasmids have identical mobilization genes (306 bp *mobC*, 972 bp *mobA* and 261 bp *mobB* located in the 3′ end of *mobA*) that belong to the HEN family of relaxases common in mobilizable plasmids in the Pasteurellaceae.^19,20^ In pB1003, a complete (804 bp) *strA* and partial (294 bp) *strB* gene are found downstream of *sul2*, and a similar gene linkage has been reported in other Pasteurellaceae plasmids.^10,21^ In pM3224T, however, there is a 711 bp *strB* gene, and the *strA* gene is disrupted by the insertion of a 568 bp element carrying *dfrA14*, a gene arrangement that has previously been reported in plasmids pCERC1 (accession no. NC_019070; Figure 1c) and pSTOJO (accession no. NG_035503) from Enterobacteriaceae isolated from humans,^22,23^ pYR1521 (accession no. NG_041026) from *Yersinia ruckeri* isolated from fish^24^ and pRSB206 (accession no. NC_025062) from an uncultured bacterium from wastewater.^25^ All of these *dfrA14-*containing plasmids share an almost identical 3 kb region from *sul2* to *strB*, although the *strB* gene is truncated (711/837 bp) in pM3224T, suggesting a common origin of this region, with recombination into the different plasmids. The insertion of *dfrA14* in a secondary site within *strA* was first noted in pUK1329 isolated from an *E. coli* of human origin in Scotland in 1995 (accession no. Z50805) but only a 681 bp fragment was sequenced. Since then, this sequence has been detected in 6.8 kb plasmids in Enterobacteriaceae of human and animal origin from around the world,^22^ as well as 5 and 53 kb plasmids in *Y. ruckeri*^24^ and an uncultured bacterium,^25^ respectively, but it has not been described in plasmids from any member of the Pasteurellaceae.

The complete nucleotide sequence of pM3389T is 6101 bp and shares greatest similarity (99% identity with 87% coverage) with pIG1 (accession no. U57647) from *P. aerogenes*^10^ and an identical plasmid found in *P. multocida* HN06^26^ (Figure 1d). These previously identified 5360 bp plasmids encode the *strA* gene upstream of *sul2*, as well as the HEN mobilization genes mentioned above, although the *mobA* gene in these plasmids is 1131 bp in length, with a 273 bp *mobB* gene encoded within the 3′ end. In pM3389T, there is an insertion of 173 bp that disrupts the end of both *mobA* and *mobB*, resulting in a 924 bp *mobA* gene with an altered 3′ end and no functional *mobB* gene. In addition, the *strA* gene is disrupted by the same 568 bp *dfrA14*-carrying element described above. However, this is the first known description of this gene arrangement upstream of *sul2*, indicating the separate recombination of just the Δ*strA*-*dfrA14-*Δ*strA* cassette instead of the entire *sul2*-Δ*strA*-*dfrA14*-Δ*strA*-*strB* region.

In both pM3224T and pM3389T, there is an 823 bp sequence upstream of *mobC* with 99% identity to the putative *oriV* originally identified in pLS88 (accession no. L23118)^27^ and common in numerous Pasteurellaceae plasmids.^21^ Although plasmids with similar *oriV* regions have been reported to replicate in *E. coli*, attempts to transform pM3224T and pM3389T into *E. coli* Stellar cells by heat shock have not been successful. It is possible that these plasmids could be transformed into *E. coli* by electroporation, but this was not investigated as isolation of the plasmids was achieved by conjugation into MIDG2331Δ*ureC*::*nadV*. A graphical analysis of the pM3224T and pM3389T sequences revealed that the region containing the *oriV* and mobilization genes has a GC content of 41%–42%, reflecting the average for Pasteurellaceae, whereas the regions containing the antimicrobial resistance genes have a GC content of 54%–55% and are likely of enterobacterial origin, as previously suggested for antimicrobial resistance genes in other Pasteurellaceae plasmids.^28^

When the complete sequences of pM3224T and pM3389T were used to search the draft genomes of the remaining trimethoprim-resistant isolates using BLASTn, contigs were identified that could be assembled into plasmids with high identity (99%–100%) to either the 6050 bp plasmid (MIDG2356 and MIDG3224) or the 6101 bp plasmid (all other trimethoprim-resistant isolates). These data indicate that the 6050 and 6101 bp plasmids have been in the UK *A. pleuropneumoniae* population since at least 1998 and 2005, respectively. The use of trimethoprim/sulphonamide combinations to treat *A. pleuropneumoniae* infection and other diseases in pigs provides selective pressure for maintenance, and the coexistence of different pathogens may facilitate the transfer of these antimicrobial resistance plasmids between different species.

In conclusion, we report here for the first time, to our knowledge, *dfrA14* in the Pasteurellaceae, which will facilitate the development of PCR assays for resistance to trimethoprim, a clinically important antimicrobial.

## Funding

This work was supported by a Longer and Larger (LoLa) grant from the Biotechnology and Biological Sciences Research Council (grant numbers BB/G020744/1, BB/G019177/1, BB/G019274/1 and BB/G018553/1), the UK Department for Environment, Food and Rural Affairs, and Zoetis (formerly Pfizer Animal Health) awarded to the Bacterial Respiratory Diseases of Pigs-1 Technology (BRaDP1T) Consortium. M. T. G. H. was supported by the Wellcome Trust (grant number 098051). The MIC work was funded from the former AHVLA's Research and Development Internal Investment Fund (grant number RD0030c). The funders had no role in study design, data collection and analysis, decision to publish or preparation of the manuscript.

## Transparency declarations

None to declare.
